# TGFβ1: An Indicator for Tumor Immune Microenvironment of Colon Cancer From a Comprehensive Analysis of TCGA

**DOI:** 10.3389/fgene.2021.612011

**Published:** 2021-04-28

**Authors:** Jinyan Wang, Jinqiu Wang, Quan Gu, Yan Yang, Yajun Ma, Quan’an Zhang

**Affiliations:** ^1^Department of Oncology, Nanjing Jiangning Hospital, The Affiliated Jiangning Hospital of Nanjing Medical University, Nanjing, China; ^2^Department of Oncology, The Affiliated Jiangning Hospital of Jiangsu Health Vocational College, Nanjing, China; ^3^Department of Oncology, Dafeng People’s Hospital, Yancheng, China; ^4^Department of Oncology, The Affiliated Cancer Hospital of Nanjing Medical University, Nanjing, China

**Keywords:** tumor-infiltrating immune cells, TGFβ1, colon cancer, indicator, tumor immune microenvironment

## Abstract

**Background:**

Tumor microenvironment (TME) and tumor-infiltrating immune cells (TICs) greatly participate in the genesis and development of colon cancer (CC). However, there is little research exploring the dynamic modulation of TME.

**Methods:**

We analyzed the proportion of immune/stromal component and TICs in the TME of 473 CC samples and 41 normal samples from The Cancer Genome Atlas (TCGA) database through ESTIMATE and CIBERSORT algorithms. Correlation analysis was conducted to evaluate the association between immune/stromal component in the TME and clinicopathological characteristics of CC patients. The difference analysis was performed to obtain the differentially expressed genes (DEGs). These DEGs were further analyzed by GO and KEGG enrichment analyses, PPI network, and COX regression analysis. Transforming growth factor β1 (TGFβ1) was finally overlapped from the above analysis. Paired analysis and GSEA were carried out to understand the role of TGFβ1 in colon cancer. The intersection between the difference analysis and correlation analysis was conducted to learn the association between TGFβ1 and TICs.

**Results:**

Our results showed that the immune component in the TME was negatively related with the stages of CC. GO and KEGG enrichment analysis revealed that 1,110 DEGs obtained from the difference analysis were mainly enriched in immune-related activities. The intersection analysis between PPI network and COX regression analysis indicated that TGFβ1 was significantly associated with the communication of genes in the PPI network and the survival of CC patients. In addition, TGFβ1 was up-regulated in the tumor samples and significantly related with poor prognosis of CC patients. Further GSEA suggested that genes in the TGFβ1 up-regulated group were enriched in immune-related activities and the function of TGFβ1 might depend on the communications with TICs, including T cells CD4 naïve and T cells regulatory.

**Conclusion:**

The expression of TGFβ1 might be an indicator for the tumor immune microenvironment of CC and serve as a prognostic factor. Drugs targeting TGFβ1 might be a potential immunotherapy for CC patients in the future.

## Background

Colon cancer (CC) is one of the most common causes of cancer-associated mortality in the United States (Goding Sauer et al., 2020). Although the overall incidence and mortality continue to decline, the incidence in young and middle-aged adults keeps rising (Siegel and Miller, 2020). Although considerable efforts have been made to improve the clinical outcomes of CC patients, CC is still a fatal disease (Zhang et al., 2019; Pan et al., 2020). Additionally, curative effect of multiple treatments, including chemotherapy, immunotherapy, and targeted therapy, are obviously reduced by drug resistance (Gao Q. et al., 2020). Hence, it is urgent to further explore the detailed molecular mechanism of CC and to identify the vital prognosis biomarkers of CC.

Recently, accumulating research has been focusing on understanding the role of the tumor microenvironment (TME) in the genesis and development of cancers. The TME is composed of multiple immune cells, stromal cells, extracellular matrix, and kinds of cytokines and chemokines (Xu et al., 2019). These components in the TME are in a dynamic process, greatly participate in tumor growth, invasion, metastasis, and drug resistance (Li and Wang, 2020; Mikami et al., 2020; Wang S. et al., 2020; Yang et al., 2020). The activation of tumor-infiltrating immune cells is an important parameter that acts as a prognostic biomarker and affects various tumor biological processes (Hu C. et al., 2020; Zhu et al., 2020). For instance, CD8-positive (CD8+) tumor-infiltrating lymphocytes (TILs) in the peri-tumoral microenvironment are significantly correlated with poor clinical outcome of salivary gland carcinoma patients (Kesar et al., 2020). Mechanically, interleukin-38 advances tumor growth by affecting CD8^+^ TILs in the TME of lung cancer (Kinoshita et al., 2020). In addition, dual suppression of both PI3K-γ and colony stimulating factor-1/colony stimulating factor-1 receptor (CSF-1/CSF-1R) pathways in tumor associated macrophages (TAM) could remodel tumor immune microenvironment (TIME) and synergistically activate antitumor immune responses in pancreatic cancer (Li et al., 2020).

In the past 10 years, tumor-infiltrating immune cells (TICs) have emerged as potential therapeutic targets. The novel therapeutic strategy, known as immune checkpoint inhibition, focuses on inhibiting the molecular communication between tumor cells and immune cells. Cytotoxic T-lymphocyte-associated protein 4 (CTLA-4) and programmed cell death protein 1 (PD-1), commonly expressed on activated T-cells, have been recognized as the most reliable targets for the immunological therapy of multiple cancers (Rotte, 2019; Liu and Zheng, 2020; Yu et al., 2020). According to the abundant clinical trials, PD-1 blocker alone, or combined with CTLA-4 have been proven to have good curative effect in various cancer types, including CC (Seidel et al., 2018; Guo et al., 2020). However, there is still a significant proportion of cancer patients who do not respond or initially respond and later develop tumor progression, indicating the existence of immune resistance (Gao A. et al., 2020). Fortunately, research has suggested that the TME and infiltrating immune cells are specific to different cancer types and might explain the immunotherapeutic responsiveness of cancers (Koirala et al., 2016; Jansen et al., 2019). As a result, further exploration of immune infiltration in CC TME is essential in clarifying the mechanisms underlying the progression of CC.

In this study, to investigate potential signatures for CC patients, we obtained a list of TME-related genes of prognostic value using immune/stromal scores after ESTIMATE algorithm-processing in multiple cohorts. Functional annotations and immune infiltration correlation were analyzed for significant hub genes. We hypothesized that TGFβ1 was correlated with poor prognosis, might act as an indicator for tumor immune microenvironment of CC, and potential immune therapies targeting TGFβ1 might provide new hope to colon patients.

## Materials and Methods

### Data Collection Based on TCGA

We collected the transcriptome RNA-seq profiling, the clinical data of CC tissues, and normal colon tissues from the TCGA database^1^. Ultimately, 514 CC cases (473 tumor samples and 41 normal samples) and the corresponding clinical data were included. We also selected the gene chip of CC (GSE41258) from the GEO database^2^. GSE41258 contained 186 primary CC tissues and 54 corresponding normal colon tissues.

### Calculation of Immune Score, Stromal Score, and ESTIMATE Score

R language version 3.6.3^3^ was used to analyze the proportion of immune/stromal component in TME of each tumor sample through ESTIMATE algorithm. Immune Score, Stromal Score, and ESTIMATE Score reflected the corresponding ratio of immune component, stromal component, and the sum of both in the TME. The higher score represented the larger ratio of immune/stromal component in the TME.

### Survival Analysis

Survival and survminer packages in R were used for the survival analysis. Kaplan–Meier plot and log-rank tests were conducted to evaluate the relationship between survival rates and differentially expressed genes (DEGs). *P* < 0.05 was considered to be statistically significant.

### Differential Analysis of Scores With Clinicopathological Characteristics

The differential analysis was performed by R language. Wilcoxon rank sum and Kruskal–Wallis rank sum test were based on the number of TNM stages for comparison.

### Affirmation of DEGs Between High-Score and Low-Score Groups and Heatmaps

A total of 473 tumor samples were classified into the high-score group and the low-score group relying on the comparison with the median score. Data analysis was performed by package limma in R. The fold change was calculated by log2 (high-score group/low-score group). A fold change (FC) > 1 and false discovery rate (FDR) < 0.05 were set up to screen DEGs. Heatmaps of DEGs were generated by pheatmap package in R.

### Gene Ontology (GO) and Kyoto Encyclopedia of Genes and Genomes (KEGG) Enrichment Analyses

GO and KEGG enrichment analyses of 1,110 DEGs were carried out by clusterProfiler, enrichplot, and ggplot2 packages in R. *P* < 0.05 was considered to be statistically significant.

### Protein–Protein Interaction (PPI) Network and Gene Set Enrichment Analysis

PPI network was constructed by the Search Tool for Retrieval of Interacting Genes/Proteins (STRING) database (version 11.0). Nodes with confidence of interactive relationship greater than 0.95 were applied. And the network was further reconstructed with Cytoscape of version 3.6.1. A functional profile of the gene set derived from the PPI was further retrieved by using Gene Set Enrichment Analysis (GSEA) 4.1.0. *P* < 0.05 was considered to be statistically significant.

### COX Regression Analysis

Univariate COX regression was performed by package survival in R.

### Tumor-Infiltrating Immune Cell Profile

TIC abundance profile in CC tumor samples was estimated by using CIBERSORT computational method. Finally, a total of 473 CC patients’ tumor samples were included for further analysis with *p* < 0.05.

### Correlation Analysis

Correlation analysis was carried out by using spearman’s correlation analysis.

### Analysis Process

We first downloaded the transcriptome RNA-seq profiling, the clinical data of CC tissues and normal colon tissues from the TCGA database, and gene chip of GSE41258 from GEO database. ESTIMATE algorithms were then used to analyze the proportion of immune/stromal component in the TME. Correlation analysis was carried out to evaluate the association between immune/stromal score and the clinic–pathological staging of CC Patients. The intersection analysis was used to obtain DEGs shared by immune score and stromal score. PPI network and univariate COX regression analysis were further conducted. The intersection analysis was carried out to find the DEGs which were both the top leading nodes in PPI network and the top factors of univariate COX regression. Finally, transforming growth factor β1 (TGFβ1) was obtained, and we focused on TGFβ1 and TGFβ signaling pathway component (smad and TGFβR) for the subsequent series of analysis, such as expression pattern analysis, survival analysis, clinic–pathological features correlation analysis, COX regression, GSEA, and correlation analysis with TICs.

## Results

### Scores Were Associated With the Clinic–Pathological Features of CC Patients

**FIGURE 1:**
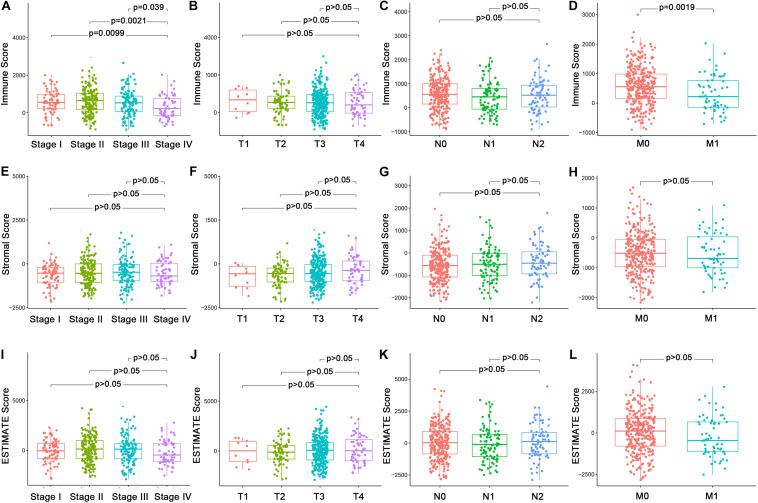
Association of Immune Score, Stromal Score, and Estimate Score with the clinic–pathological features of CC Patients. **(A–D)** Association of Immune Score with stages and TNM classification by Kruskal–Wallis rank sum test. **(E–H)** Association of Stromal Score with stages and TNM classification Kruskal–Wallis rank sum test. **(I–L)** Association of Estimate Score with stages and TNM classification Kruskal–Wallis rank sum test.

In order to explore the underlying associations between the ratio of immune/stromal components and the clinic-pathological features (Supplementary Table 1), we analyzed the TNM stages of CC patients regarding Immune Score, Stromal Score, and ESTIMATE Score (Figure 1). Interestingly, There were significant differences in the Immune Score in stage I compared with stage II, III, and IV (Figure 1A, *p* = 0.0099, 0.0021, 0.039). In particular, Immune Score was negatively related with M classification of TNM stages (Figure 1D, *p* = 0.0019). However, Stromal Score and ESTIMATE Score had nothing to do with the TNM stages of CC patients (Figures 1E–L, *p* > 0.05). The above results indicated that the proportion of immune components might play an important role in the advance of CC, especially distant metastasis.

### DEGs Shared by Immune Score and Stromal Score Were Significantly Associated With Immune-Related Activities

**FIGURE 2:**
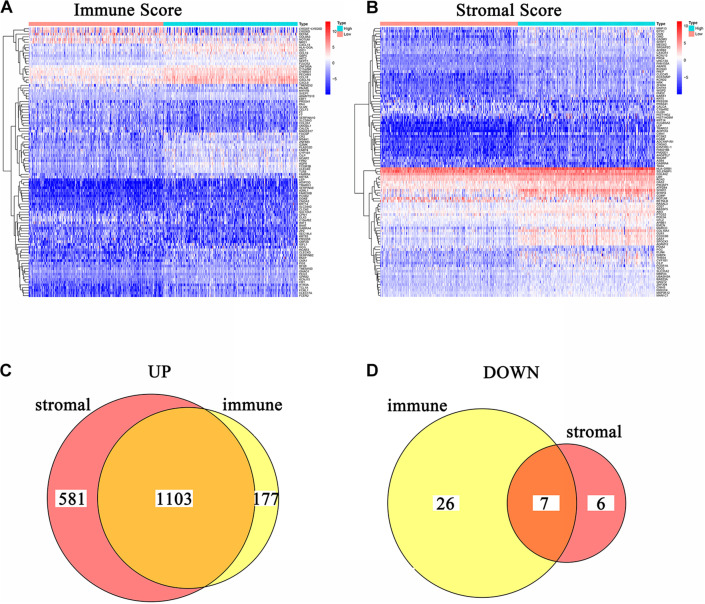
Heatmaps and Venn plots of DEGs. **(A)** Heatmap for DEGs conducted by comparing high Immune Score tumor samples with low Immune Score tumor samples. Row name: the gene name. Column name: the ID of samples which are not shown in plot. DEGs were examined by Wilcoxon rank sum test with *q* = 0.05 and log2^fold–change^ > 1 as the significance threshold. **(B)** Heatmap for DEGs conducted by comparing high Stromal Score tumor samples with low Stromal Score tumor samples. Row name: the gene name. Column name: the ID of samples which are not shown in plot. DEGs were examined by Wilcoxon rank sum test with *q* = 0.05 and log2^fold–change^ > 1 as the significance threshold. **(C,D)** Venn plots of commonly overexpressed and downexpressed DEGs shared by Immune Score and Stromal Score.

**FIGURE 3:**
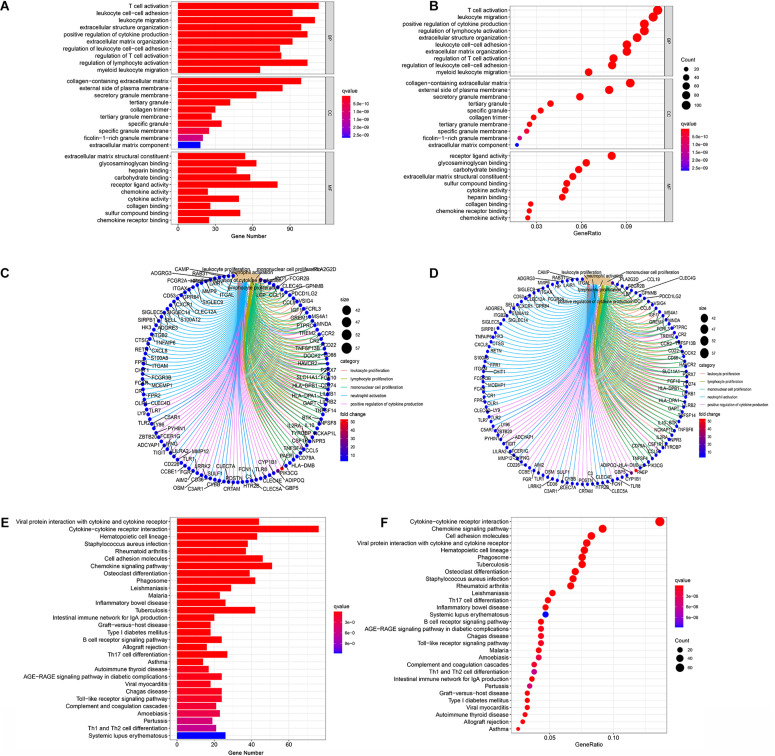
Enrichment analysis GO and KEGG for 1,110 DEGs. **(A–C)** GO enrichment analysis for 1,110 DEGs, *p* < 0.05 was considered to be enriched significantly. **(D–F)** KEGG enrichment analysis for 1,110 DEGs, *p* < 0.05 was considered to be enriched significantly.

In order to acquire the detailed gene profile in TME, the difference analysis between high score and low score tumor samples were conducted. The results displayed that a total of 1,313 DEGs were acquired from the immune score group (high score tumor samples vs. low score tumor samples), among which 1,280 DEGs were up-regulated and 33 DEGs were down-regulated when compared to the median (Figure 2A). In addition, 1,697 DEGs were acquired from the stromal score group, including 1,684 up-regulated genes and 13 down-regulated genes (Figure 2B). Furthermore, the intersection analysis was carried out to obtain the up-regulated or down-regulated genes both in the immune score and stromal score. The Venn plot displayed that 1,103 genes were up-regulated and 7 genes were down-regulated in both the immune score and stromal score (Figures 2C,D). These DEGs, a total of 1,110 genes, might play a significant role in regulating the status of the TME. Therefore, GO enrichment analysis and KEGG enrichment analysis were used to evaluate the functions of these DEGs. GO enrichment analysis revealed that these DEGs were closely associated with immune-related GO terms, including T cell activation, leukocyte migration, positive regulation of cytokine production, and so on (Figures 3A–C). Besides, KEGG enrichment analysis indicated that 1,110 DEGs were significantly related with cytokine-cytokine receptor interaction, chemokine signaling pathway, positive regulation of cytokine production, mononuclear cell proliferation and so on (Figures 3D–F). From above, the functions of these DEGs seemed to be significantly associated with immune-related activities and might be a predominant characteristic of the TME in CC.

### Intersection Analysis Between the PPI Network and Univariate COX Regression

**FIGURE 4:**
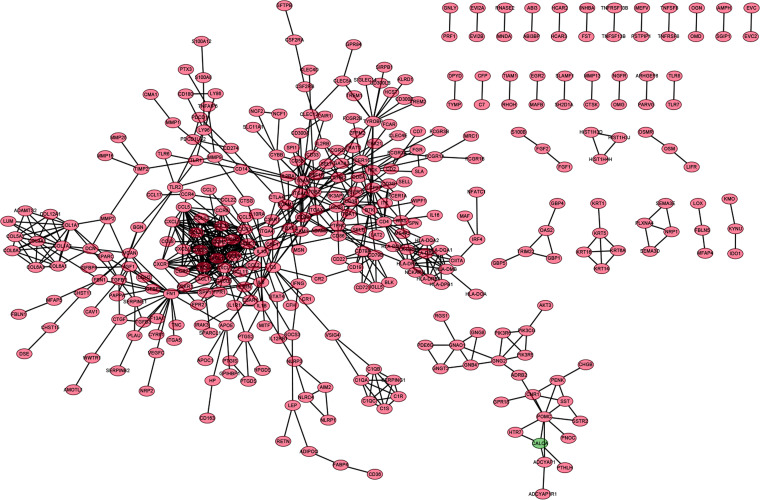
PPI network of DEGs. PPI network was carried out with the nodes with interaction confidence value > 0.95.

**FIGURE 5:**
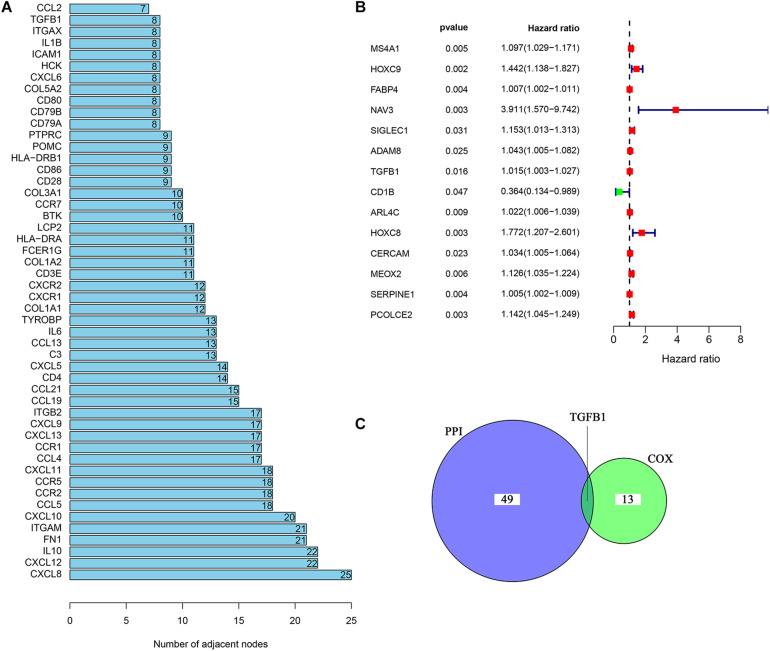
Univariate COX regression analysis of DEGs. **(A)** The top 50 genes ranked by the number of nodes. **(B)** Univariate COX regression analysis of DEGs. *p* < 0.05 was considered as the significance threshold. **(C)** Venn plot displays the common genes shared by the top 50 genes in PPI and top significant genes in univariate COX regression analysis.

In order to further explore the underlying mechanisms regarding these 1,110 DEGs, we first constructed the PPI network by using STRING database and Cytoscape software. The detailed interactions between 1,110 DEGs were displayed in Figure 4. The top 50 DEGs ranked by the number of nodes were shown in Figure 5A. Univariate COX regression analysis was conducted to find the most significant DEGs regarding the survival of CC patients (Figure 5B). Finally, the intersection analysis was carried out to find the DEGs which were both the top 50 leading nodes in the PPI network and the top 14 factors of the univariate COX regression. Transforming growth factor β1 (TGFβ1) was overlapping from the above analysis (Figure 5C).

### TGFβ1 Was Associated With the Survival and Clinic–Pathological Staging of CC Patients

**FIGURE 6:**
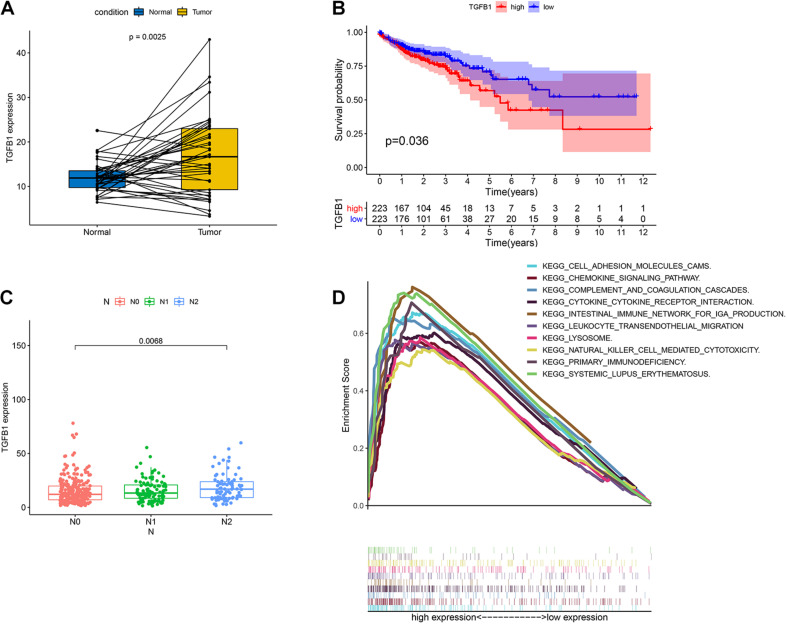
The expression of TGFβ1 in CC patients and the association with survival, TNM classification and GSEA. **(A)** Paired differentiation analysis for expression of TGFβ1 in the colon cancer samples and the paired normal samples deriving from the same patient (*p* = 0.0025 by Wilcoxon rank sum test). **(B)** Survival analysis for colon cancer patients with high expression or low expression of TGFβ1. *p* = 0.036 by log-rank test. **(C)** Association of the expression of TGFβ1 with N stage by Kruskal–Wallis rank sum test; *p* = 0.0068. **(D)** GSEA for tumor samples with high expression or low expression of TGFβ1.

TGFβ1 was a pleiotropic cytokine and played a vital role in immune reconstruction (Chen et al., 2012; Kövy et al., 2020). Teixeira et al. (2011) discovered that TGFβ1 affected non-small cell lung cancer (NSCLC) susceptibility with impact in cellular microenvironment. In our study, the pairing analysis revealed that TGFβ1 was up-regulated in the tumor samples compared to that in the paired normal samples from the same patients (Figure 6A, *p* = 0.0025). We then divided CC samples into two groups, including TGFβ1 high-expression group and TGFβ1 low-expression group. The survival analysis revealed that CC patients with up-regulated TGFβ1 had shorter survival than that of down-regulated TGFβ1 (Figure 6B, *p* = 0.036). Specifically, the up-regulated TGFβ1 was related to the lymph node stage of CC patients (Figure 6C). In conclusion, TGFβ1 was negatively associated with the prognosis of CC patients.

### TGFβ1 Might Participate in the Modulation of the TME

Considering that the expression of TGFβ1 was negatively associated with the survival and lymph node stages of CC patients, GSEA was carried out in the up-regulated and the down-regulated groups compared with the median level of TGFβ1 expression, respectively. The genes in TGFβ1 up-regulated group were primarily enriched in immune-related activities, including cell adhesion molecules, chemokine signaling pathway, complement and coagulation, cytokine-cytokine receptor interaction and so on (Figure 6D). Nonetheless, few genes were enriched in the TGFβ1 down-regulated group. The above results indicated that TGFβ1 might participate in the modulation of TME.

### TGFβ1 Signaling Pathway Was Associated With TICs in TIME

**FIGURE 7:**
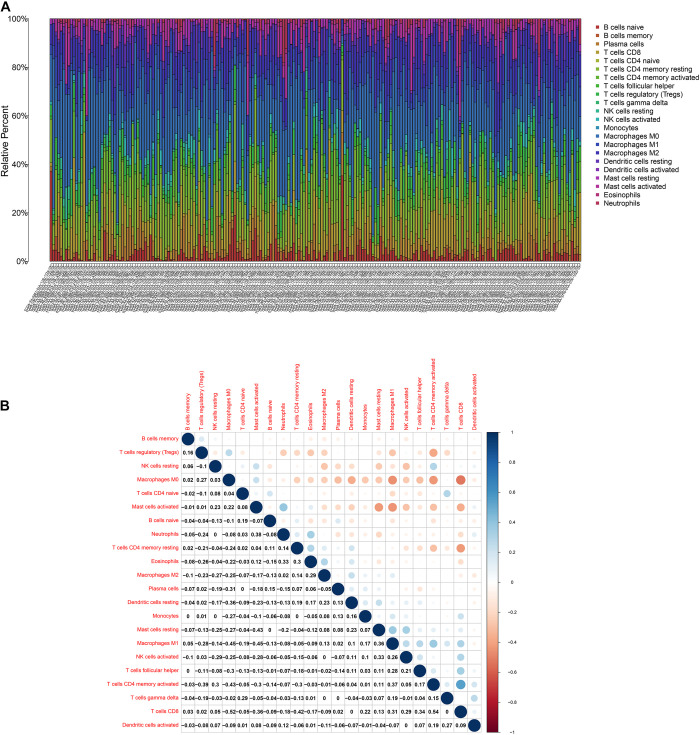
TIC profile. **(A)** Barplot displays the ratio of 22 kinds of TICs in colon cancer samples. Column names: sample ID. **(B)** Heatmap displays the association between 22 kinds of TICs; each spot represents the *p*-value of correlation between two kinds of cells; Pearson coefficient was carried out for significance test.

**FIGURE 8:**
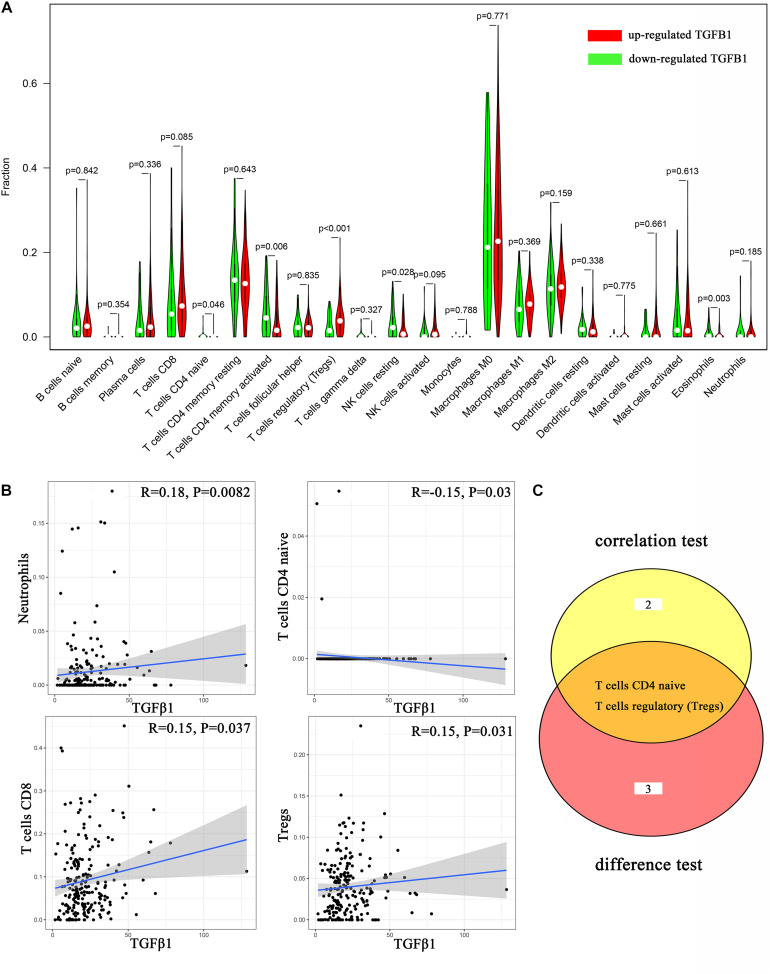
Correlation analysis between TICs and TGFβ1. **(A)** Violin plot displays the differentiation of 22 kinds of TICs between colon cancer samples with high or low expression of TGFβ1; Wilcoxon rank sum was carried out for the significance test. **(B)** Scatter plot displays the association between 4 kinds of TICs and the expression of TGFβ1; Pearson coefficient was carried out for the correlation test; *p* < 0.05 was considered as the significance threshold and plotted. **(C)** Venn plot displays two kinds of TICs shared by difference and correlation tests showed in violin and scatter plots, respectively.

**FIGURE 9:**
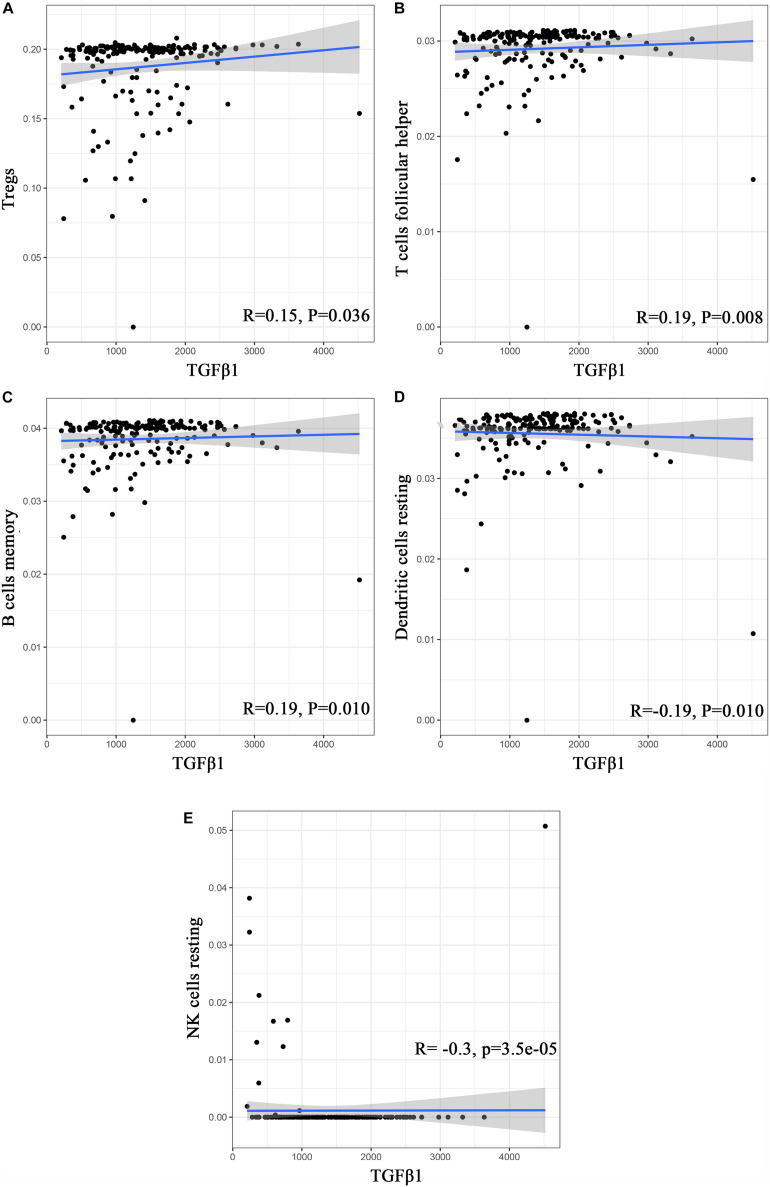
Correlation analysis between TICs and TGFβ1 in the gene chip of GSE41258. **(A–E)** Scatter plot displays the association between 4 kinds of TICs and the expression of TGFβ1; Pearson coefficient was carried out for the correlation test; *p* < 0.05 was considered as the significance threshold and plotted.

To further affirm the connection between TGFβ1 and the immune microenvironment, we applied CIBERSORT algorithm to examine the ratio of tumor-infiltrating immune cells in CC (Figures 7A,B). The difference test indicated that 5 kinds of TICs were significantly related with the expression of TGFβ1, such as T cells CD4 naïve, T cells CD4 memory activated, Tregs, NK cells resting, and eosinophils (Figure 8A). The correlation test revealed that neutrophils, Tregs, and T cells CD8 were positively related with the expression of TGFβ1, and T cells CD4 naïve was negatively related with the expression of TGFβ1 (Figure 8B). The intersection between the difference test and correlation test suggested that T cells CD4 naïve and Tregs were potentially associated with the expression of TGFβ1 (Figure 8C and Supplementary Table 2). In order to further confirm the correlation between TGFβ1 and TICs in TIME of CC, we conducted correlation analysis by using the data in GSE41258. The result indicated that several TICs, including B cells memory, T cells follicular helper, Tregs, NK cells resting, and dendritic cells resting, were significantly correlated with the expression level of TGFβ1 (Figure 9). From the above, we speculated that TGFβ1 might closely communicate with Tregs to greatly participate in the immune activities of the TIME in CC.

In addition, it was known that TGFβ1 participated in multiple biological processes through binding to TGFβR, including TGFβR1, TGFβR2, and TGFβR3 (Wang et al., 2019). We conducted correlation analysis between TGFβR and TICs in CC and found out that TGFβR took a great part in communicating with TICs in the TIME of CC (Table 1). In addition, smad proteins were critical downstream mediators of TGFβ signaling pathway and regulated the transcription of direct target genes of TGF-β (Luo, 2017). Corresponding correlation analysis was also conducted to evaluate the relationship between smad and TICs in the TIME of CC. The result showed that smad1-7, 9 were significantly associated with various TICs in the TIME of CC (Table 2). In conclusion, not only TGFβ1, but also TGFβ signaling pathway components were closely related with the TIME of CC, partly through communicating with multiple TICs. These results further indicated the potential role of TGFβ1 in the immune activity of the TME.

**TABLE 1:** Correlation analysis between TGFβR and TICs in TIME of CC.

TGFβ R	TICs	*P*–value
TGFβR1	B cells naive	0.040933844
	T cells CD8	8.39E-07
	T cells CD4 memory resting	0.000373437
	T cells regulatory (Tregs)	0.020476451
	NK cells activated	0.004390418
	Neutrophils	0.00519773
TGFβR2	B cells naive	0.045588132
	B cells memory	0.010537954
	Plasma cells	0.009197408
	T cells CD8	2.18E-07
	T cells CD4 memory resting	0.000106305
	T cells CD4 memory activated	0.002443409
	T cells follicular helper	0.002419206
	NK cells activated	2.85E-08
	Macrophages M0	0.013539433
	Macrophages M1	0.012813639
TGFβR3	B cells memory	0.00405551582282848

*p > 0.05 was not displayed.*

**TABLE 2:** Correlation analysis between SMAD and TICs in TIME of CC.

SMAD	TICs	*P*-value
SMAD1	B cells naive	0.021093
	T cells CD4 memory resting	2.83E-06
	T cells regulatory (Tregs)	0.005022
	NK cells activated	0.010779
	Macrophages M1	0.021773
	Mast cells resting	0.018896
SMAD2	T cells CD4 memory resting	0.001936
	T cells follicular helper	0.020209
	T cells regulatory (Tregs)	4.25E-06
	Macrophages M0	0.000262
	Macrophages M1	0.04713
	Dendritic cells activated	0.004243
	Neutrophils	0.025751
SMAD3	T cells follicular helper	0.027115
	T cells gamma delta	0.032897
	Macrophages M2	0.041467
SMAD4	Plasma cells	0.012128
	T cells CD8	0.022337
	T cells CD4 memory activated	0.020959
	T cells follicular helper	0.003017
	T cells regulatory (Tregs)	7.93E-05
	Macrophages M0	0.000796
	Macrophages M1	0.002791
	Neutrophils	0.036174
SMAD5	B cells naive	0.000237
	T cells CD8	0.0007
	T cells CD4 memory resting	1.38E-09
	T cells regulatory (Tregs)	3.15E-06
	NK cells activated	0.029976
SMAD6	T cells CD8	0.047908
	T cells regulatory (Tregs)	2.54E-06
SMAD7	T cells CD8	0.021056
	T cells CD4 memory activated	8.65E-06
	T cells regulatory (Tregs)	6.54E-06
	NK cells activated	9.27E-05
	Macrophages M0	3.47E-06
	Macrophages M1	0.003951
SMAD9	B cells naive	0.000231
	T cells CD8	2.67E-07
	T cells CD4 naive	0.048259
	T cells CD4 memory resting	5.00E-08
	T cells CD4 memory activated	9.27E-05
	T cells follicular helper	0.003673
	NK cells activated	0.021077
	Macrophages M1	0.039982
	Macrophages M2	0.026902

*p > 0.05 was not displayed.*

## Discussion

In recent years, great advances have been made in the exploration of the CC treatment. It gradually moves away from chemotherapy, which has been the standard treatment of CC for decades, toward immunotherapy that modulates immune responses against tumor cells (Franke et al., 2019; Chalabi et al., 2020). In the last few years, the TME was found to play a vital role in the initiation and progression of tumorigenesis (Corn et al., 2020; Uttam et al., 2020; Wang J. et al., 2020; Zhang Z. et al., 2020). Research has indicated that the TIME was closely related with the prognosis of cancers, including hepatocellular carcinoma (Hu Z.Q. et al., 2020), lung adenocarcinoma, lung squamous cell carcinoma (Zhang L. et al., 2020), and so on. Therefore, it is of great benefit to identify the vital potential biomarkers associated with the remodeling of the TME and prognosis, and develop more specific drugs against CC.

In this study, we attempted to comprehensively assess the TME of CC and identify TME-associated genes that related with the survival and clinic–pathological features of CC patients from the TCGA database. Firstly, we evaluated the proportion of the immune/stromal component in the TME and found out that the immune/stromal component was associated with the clinic–pathological staging of CC patients. Secondly, DEGs, shared by immune score and stromal score, were found out and further explored by the PPI network and univariate COX regression analysis. Thirdly, TGFβ1 came to our eyes and we carried out survival analysis, clinic–pathological features correlation analysis, COX regression, GSEA, and TICs correlation analysis. Finally, based on the above research, we concluded that TGFβ1 and TGFβ signaling pathway components played a significant role in the TIME of CC potential through communicating with various TICs. TGFβ1 might be a potential biomarker for the status of the TIME in CC patients.

In Chen et al. (2017) carried out a meta-analysis and found out that up-regulated TGF-β had a favorable impact on overall survival (OS) and disease-free survival (DFS) in CC patients and might be used as a prognostic biomarker for CC patients undergoing surgery. Later, Zessner-Spitzenberg et al. (2019) also concluded that elevated levels of TGFβ served as a poor prognostic biomarker of CC. The TGFβ family included three isoforms, TGFβ1, TGFβ2, and TGFβ3, with TGFβ1 being the most prominent (Clark and Coker, 1998). TGFβ1 was a multi-functional cytokine and regulated a variety of biologic processes in the host, such as cell proliferation, apoptosis, differentiation, migration, invasion and angiogenesis (Massagué, 2000). In addition, it was aberrantly activated in the late-stages of tumorigenesis and the dysregulation of TGFβ1 signaling pathway contributed to various aspects of cancer progression (Zarzynska, 2014; Tang et al., 2018). Recently, studies indicated that TGFβ1 greatly participated in numerous immune regulatory functions, such as tumor immune suppression and escape (Hargadon, 2016). It was also significantly associated with the function of kinds of TICs, such as tumor-associated macrophages (Machado et al., 2014), dendritic cells (Laouar et al., 2008; Ramalingam et al., 2012), neutrophils (Fan et al., 2014), T cells (Chou et al., 2012), and natural killer cells (Viel et al., 2016), which might predict the prognosis in the treatment of CC (Galon et al., 2006). However, no further research was carried out to evaluate the relationship between TGFβ1 and TICs in regulating the TIME of CC. In our research, we found that TGFβ1 and TGFβ signaling pathway components might play a significant role in the TIME of CC partly through communicating with various TICs, and TGFβ1 might be a potential biomarker for the status of the TIME in CC patients. Further research might focus on the complicated communications between TGFβ1 and specific TICs in modulating the TIME of CC.

## Conclusion

Our study implied that TGFβ1 greatly participated in the modulation of the colon cancer TIME through communicating with T cells CD4 naïve and Tregs. Furthermore, TGFβ1 might be a potential indicator of the status of the TIME in CC patients. Therefore, further investigations are needed to clarify the detailed mechanisms among TGFβ1, T cells CD4 naïve, and Tregs. Drugs targeting TGFβ1 might be a potential treatment for CC patients in the future.

## Data Availability Statement

The datasets presented in this study can be found in online repositories. The names of the repository/repositories and accession number(s) can be found in the article/Supplementary Material.

## Author Contributions

JyW and QZ: conceptualization, writing—review, and editing. JqW: methodology and investigation. QG: software, resources, and data curation. QZ: validation. JyW: formal analysis. JyW, JqW, and QZ: writing—original draft preparation. YY: supervision. YM: project administration. JyW, YY, and QZ: funding acquisition. All authors have read and agreed to the published version of the manuscript.

## Conflict of Interest

The authors declare that the research was conducted in the absence of any commercial or financial relationships that could be construed as a potential conflict of interest.

## Footnotes

1 https://portal.gdc.cancer.gov/

2 https://www.ncbi.nlm.nih.gov/

3 https://www.r-project.org/

## Supplementary Material

Supplementary Table 1 The clinic–pathological features of CC Patients from TCGA database.

Supplementary Table 2 The intersection between the difference test and correlation test.
