# Supplementary material for: TGFβ1: An Indicator for Tumor Immune Microenvironment of Colon Cancer From a Comprehensive Analysis of TCGA
Source: Front Genet. 2021 Apr 28;12:612011. doi: 10.3389/fgene.2021.612011 (PMC8115728; doi:10.3389/fgene.2021.612011)
Supplement: Supplementary Table 1 — The clinic–pathological features of CC Patients from TCGA database. [file Table_1.docx]

Supplement Table 1: The clinic–pathological features of CC Patients from TCGA database.

| Clinic–pathological features |  | Total | % |
| --- | --- | --- | --- |
| Age | <=65 | 184 | 40.80 |
|  | >65 | 267 | 59.20 |
| Gender | Male | 237 | 52.55 |
|  | Female | 214 | 47.45 |
| Stage | I | 75 | 16.63 |
|  | II | 178 | 39.47 |
|  | III | 125 | 27.72 |
|  | IV | 62 | 13.75 |
|  | Unknown | 11 | 2.44 |
| T Classification | T1 | 10 | 2.22 |
|  | T2 | 77 | 17.07 |
|  | T3 | 308 | 68.29 |
|  | T4 | 56 | 12.42 |
| N Classification | N0 | 268 | 59.42 |
|  | N1 | 103 | 22.84 |
|  | N2 | 80 | 17.74 |
| M Classification | M0 | 334 | 74.06 |
|  | M1 | 62 | 13.75 |
|  | Unknown | 55 | 12.20 |
