# Supplementary material for: TGFβ1: An Indicator for Tumor Immune Microenvironment of Colon Cancer From a Comprehensive Analysis of TCGA
Source: Front Genet. 2021 Apr 28;12:612011. doi: 10.3389/fgene.2021.612011 (PMC8115728; doi:10.3389/fgene.2021.612011)
Supplement: Supplementary Table 2 — The intersection between the difference test and correlation test. [file Table_2.docx]

Supplement Table 2: The intersection between the difference test and correlation test.

| TICs | Correlation test (p value) | Difference test (p value) |
| --- | --- | --- |
| T cells CD4 naive | 0.030 | 0.046 |
| T cells CD4 memory activated | >0.05 | 0.006 |
| T cells regulatory (Tregs) | 0.030 | 0.001 |
| NK cells resting | >0.05 | 0.028 |
| Eosinophils | >0.05 | 0.003 |
| T cells CD8 | 0.036 | >0.05 |
| Neutrophils | 0.008 | >0.05 |
